# Different Effects of Different *Lactobacillus acidophilus* Strains on DSS-Induced Colitis

**DOI:** 10.3390/ijms232314841

**Published:** 2022-11-27

**Authors:** Zheng Huang, Lei Gong, Yan Jin, Catherine Stanton, Reynolds Paul Ross, Jianxin Zhao, Bo Yang, Wei Chen

**Affiliations:** 1 State Key Laboratory of Food Science and Technology, Jiangnan University, Wuxi 214122, China; 2 School of Food Science and Technology, Jiangnan University, Wuxi 214122, China; 3 Department of Gastroenterology, The Affiliated Wuxi Second People’s Hospital of Nanjing Medical University, Wuxi 214122, China; 4 International Joint Research Laboratory for Pharmabiotics & Antibiotic Resistance, Jiangnan University, Wuxi 214122, China; 5 APC Microbiome Ireland, University College Cork, T12 K8AF Cork, Ireland; 6 Teagasc Food Research Centre, Moorepark, Fermoy, P61 C996 Co. Cork, Ireland; 7 National Engineering Research Center for Functional Food, Jiangnan University, Wuxi 214122, China

**Keywords:** *Lactobacillus acidophilus*, colitis, cytokines, gut microbiota, SCFAs

## Abstract

Inflammatory bowel disease (IBD) is a worldwide chronic intestinal inflammatory immune-related disease. In this study, mice with dextran sulfate sodium (DSS)-induced colitis were used to evaluate the effect of *Lactobacillus acidophilus* on colitis. The results revealed that *L. acidophilus* CCFM137 and FAHWH11L56 show potential for relieving colitis symptoms, while *L. acidophilus* FGSYC48L79 did not show a protective effect. Moreover, *L. acidophilus* NCFM and FAHWH11L56 showed similar effects on various indicators of DSS-induced colitis, increasing the IL-10 and IL-17 in the colon, and modifying the CCL2/CCR2 axis and CCL3/CCR1 axis. For *L. acidophilus* CCFM137, its effects on colitis were different from the above two strains. Moreover, *L. acidophilus* FGSYC48L79 had negative effects on colitis by increasing the abundance of harmful bacteria in the gut microbiota and may promote the signaling of chemokines and their receptors. This may be related to its special genome compared to the other strains.

## 1. Introduction

Inflammatory bowel disease (IBD) is a chronic intestinal inflammatory disease. IBD has evolved into a global healthcare problem, although its etiology remains unknown, which might include genetics, environment, and microbial factors [1]. However, long-term use of medicines to relieve colitis will cause some side effects. In the past decade, probiotics, synbiotics, and postbiotics have become alternative therapies for IBD treatment. These “probiotics-related” products could promote human immunity directly, stimulate the human body to generate some healthy secondary metabolites, lay a solid foundation for the invasion of pathogenic bacteria, and keep the host in a microecological balance situation.

*L. acidophilus* can be found in the human gastrointestinal tract, oral cavity, and vagina. Generally regarded as safe and edible bacteria, *L. acidophilus* has been found to have some positive properties for gastrointestinal health, such as regulating gut microbiota [2], alleviating diarrhea [3], and relieving colitis [4]; many *L. acidophilus* strains can alleviate colitis by regulating the secretion of cytokines in the intestine, improving the intestinal barrier, and/or regulating the production of SCFAs [5,6,7]. As one of the most famous probiotic strains, *L. acidophilus* NCFM has also been reported to have the potential to relieve colitis [8].

In our previous research on the comparative genomics of *L. acidophilus*, *L. acidophilus* NCFM, CCFM137, FAHWH11L56, and FGSYC48L79 which were isolated from human intestine, it was found that they have some genetic differences [9]. At present, no research has connected the genetic differences and functional differences on colitis of *L. acidophilus*. Hence, this study aimed to assess the effect of *L. acidophilus* strains with genetic differences on DSS-induced colitis and to determine the potentially different mechanisms for affecting colitis.

## 2. Results

### 2.1. L. acidophilus Improved the Symptoms of Mice with Colitis

**Figure 1:**
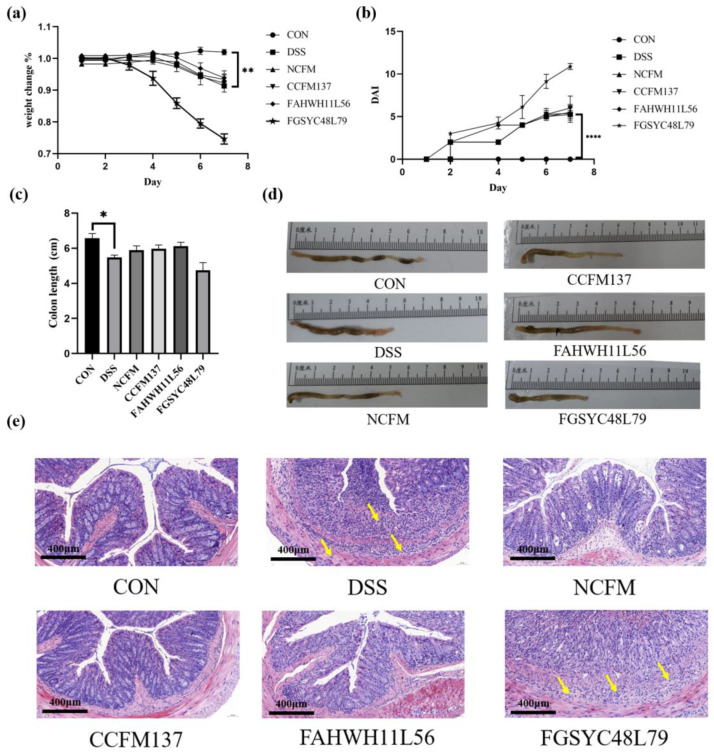
Effect of *L. acidophilus* on symptoms of colitis. (**a**) Body weight, (**b**) disease activity index (DAI), (**c**) colon length, (**d**) macroscopic pictures of colons (The definition of the Chinese term in the figure is centimeter), and (**e**) colon morphology. *: *p* < 0.05. **: *p* < 0.01. ****: *p* < 0.0001. All data are presented as mean ± standard error of the mean (SEM), The magnification is 20×, the yellow arrows represent lymphocyte and centriocyte infiltration.

A mice model with colitis caused by DSS was established and, during the DSS exposure, the body weight and DAI of each mouse were recorded daily. Compared with the initial situation, the body weight of DSS-challenged mice decreased significantly (8.8% weight loss) and the DAI increased significantly (*p* < 0.05), while the mice in the control group had no negative changes in these two indexes. The treatment of *L. acidophilus* NCFM (6.6% weight loss), *L. acidophilus* CCFM137 (7.7% weight loss), and *L. acidophilus* FAHWH11L56 (6.0% weight loss) did not significantly change the mice’s body weight. Conversely, in the *L. acidophilus* FGSYC48L79 group, the average body weight of mice was 25.5% lower than their initial average body weight (Figure 1a,b). In the control group, the colon length was 6.57 ± 0.76 cm, and their colon was normal reddish and contained granular feces (Figure 1d). In comparison, the average colon length in the DSS group was 5.4 ± 0.26 cm with more watery contents (Figure 1c,d). Compared with the control group, DSS exposure resulted in an 18.0% reduction in colon length. The colon lengths of mice treated with *L. acidophilus* NCFM, CCFM137, and *L. acidophilus* FAHWH11L56 were 5.89 ± 0.69 cm, 5.98 ± 0.60 cm, and 6.12 ± 0.63 cm, respectively (Figure 1c). Additionally, similar to the changes in body weight and DAI in the *L. acidophilus* FGSYC48L79 group, the average colon length of mice was 4.75 ± 1.23 cm, and the colon shortening was 9.7% higher than that in the DSS group (Figure 1a–c).

To evaluate the intestinal injury, H&E staining was performed. The results showed that, in the control group, the mucosal layer of the intestinal epithelium was intact, the epithelial cell morphology and structure were normal, the lamina propria intestinal glands were in a normal condition, the goblet cells were abundant, and there was no obvious inflammatory cell infiltration in the colons of control mice. After DSS exposure, ulcers could be seen in the mucosal layer, and the intestinal epithelium was destroyed. Additionally, the lamina propria intestinal glands were necrotic and dissolved, replaced by hyperplastic connective tissue. Meanwhile, a small amount of connective tissue could be seen to have proliferated to the submucosa; more lymphocytes and the center could be seen in the lamina propria and submucosa granulocyte infiltration. At the same time, individual inflammatory cells infiltrated into the muscle layer, and more necrotic cell fragments could be seen in the intestinal lumen. To a certain extent, administering *L. acidophilus* NCFM, CCFM137, and FAHWH11L56 could restore the intestinal epithelial structure and reduce edema and inflammatory infiltration, while *L. acidophilus* FGSYC48L79 treatment aggregated the intestinal damage by DSS (Figure 1e).

### 2.2. L. acidophilus Affected the Cytokines in the Colon of Mice

**Figure 2:**
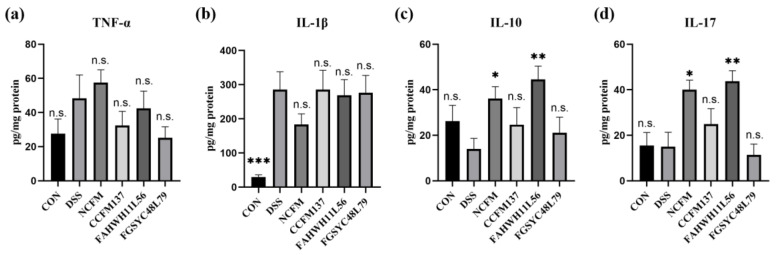
Effect of *L. acidophilus* on cytokines in colon. (**a**) TNF-α, (**b**) IL-1β, (**c**) IL-10, and (**d**) IL-17. *: *p* < 0.05. **: *p* < 0.01. ***: *p* < 0.001. N.s.: no significant difference. All data are presented as mean ± standard error of the mean (SEM). The DSS group was used as a reference.

The concentrations of IL-1β, IL-10, IL-17, and TNF-α in the colon were analyzed by ELISA to assess the effects of *L. acidophilus* on inflammatory cytokines. The results showed that TNF-α in the colon was not changed significantly after DSS challenge or *L. acidophilus* treatment (Figure 2a). For IL-1β, after DSS exposure, its concentration in the colon increased significantly (*p* < 0.05), and *L. acidophilus* did not adjust IL-1β significantly (Figure 2b). Compared with the control group, the contents of IL-10 and IL-17 in the colon of mice in the DSS group showed no significant changes, although the interventions of *L. acidophilus* NCFM and FAHWHLL156 increased the concentration of IL-10 and IL-17 significantly, compared with the DSS group (*p* < 0.05). However, the intervention of *L. acidophilus* CCFM137 and FGSYC48L79 had no significant effect on IL-10 and IL-17 (Figure 2c,d).

### 2.3. L. acidophilus Affected the CCL2/CCR2 Axis and CCL3/CCR1 Axis Downstream IL-17 Signal Pathway in the Colon of Mice

**Figure 3:**
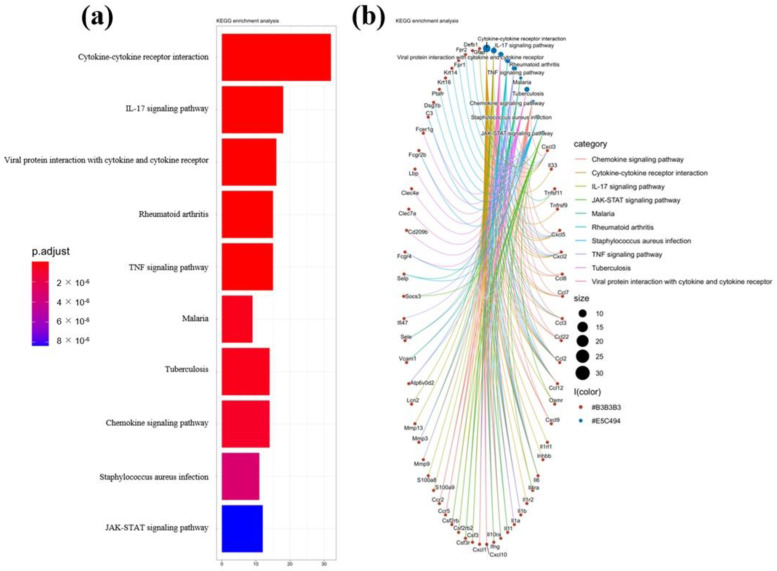
KEGG enrichment analysis in colon of mice with DSS-induced colitis. (**a**) Histogram of KEGG enrichment analysis, (**b**) Network Diagram of KEGG enrichment analysis.

**Figure 4:**
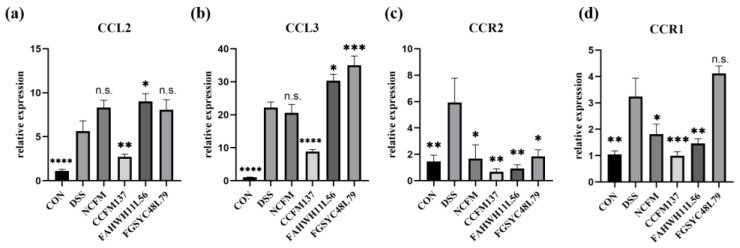
Effects of *L. acidophilus* on chemokines and their receptors in colon. (**a**) CCL2, (**b**) CCL3, (**c**) CCR2, and (**d**) CCR1. *: *p* < 0.05. **: *p* < 0.01. ***: *p* < 0.001. ****: *p* < 0.0001. N.s.: no significant difference. All data are presented as mean ± standard error of the mean (SEM). The DSS group was used as a reference.

By analyzing the GSE22307 series of the GEO database, it was found that the IL-17 pathway was one of the significantly changed pathways in mice with DSS-induced colitis, and the significantly changed genes included a variety of cellular chemokines and their receptors (Figure 3). To explore how *L. acidophilus* affected colitis by regulating IL-17, the downstream-related chemokines, CCL2 and CCL3, and their corresponding receptors, CCR2 and CCR1, were further analyzed in the IL-17 signaling pathway. The expression of CCL2 and CCL3 in the colon of the mice in the DSS group was significantly higher than in the control group (*p* < 0.05). In *L. acidophilus* groups, only *L. acidophilus* CCFM137 significantly down-regulated the relative expression of CCL2 and CCL3 in the colon (*p* < 0.05), whereas the other three strains showed no significant change, and even further up-regulated the expression of CCL2 and CCL3 in the colon (Figure 4a,b).

For CCR2 and CCR1, their relative expression levels were significantly increased after DSS exposure, while the intervention with *L. acidophilus* NCFM, CCFM137, and FAHWH11L56 significantly decreased the expression levels of CCR2 and CCR1 (*p* < 0.05). The effect of *L. acidophilus* FGSYC48L79 on CCR2 was consistent with the other *L. acidophilus* strains, although, except for CCR1, it did not show a significant effect (*p* > 0.05) (Figure 4c,d).

### 2.4. L. acidophilus Modified the Diversity of Gut Microbiota in DSS-Induced Colitis Mice

**Figure 5:**
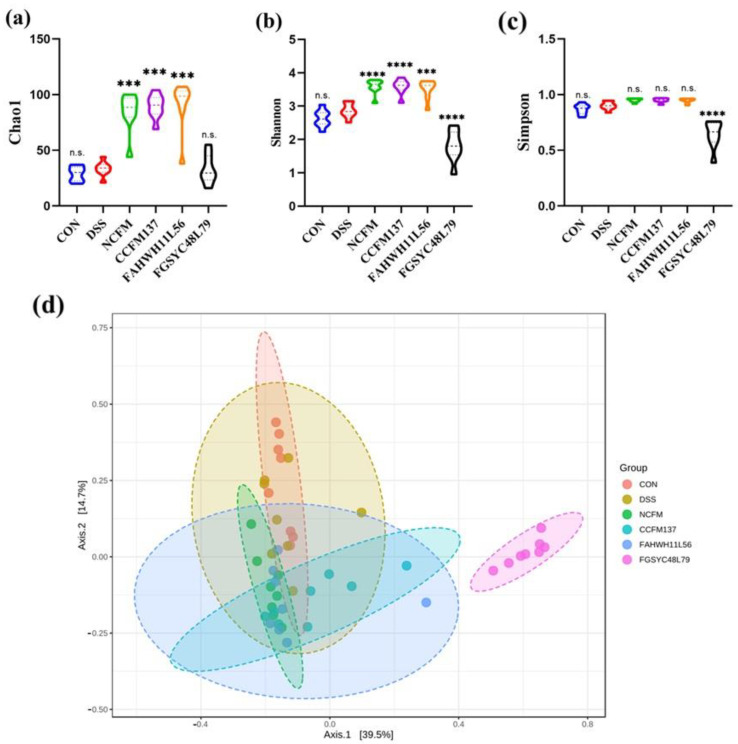
Effects of *L. acidophilus* on the diversity of gut microbiota. (**a**–**c**) Chao1, Shannon, and Simpson indexes of gut microbiota. (**d**) Beta diversity of gut microbiota. ***: *p* < 0.001. ****: *p* < 0.0001. N.s.: no significant difference. All data are presented as mean ± standard error of the mean (SEM). The DSS group was used as a reference.

To assess the effect of *L. acidophilus* on gut microbiota, 16S rRNA amplicon sequencing was used to analyze the gut microbiota. The α-diversity of gut microbiota was reflected by Chao1, Shannon, and Simpson indexes. For the Chao 1 and Shannon indexes, there was no significant difference (*p* > 0.05) between control and the DSS group; however, *L. acidophilus* NCFM, CCFM137, and FAHWH11L56 interventions increased both indexes significantly (*p* < 0.05), while *L. acidophilus* FGSYC48L79 significantly decreased the Shannon index (*p* < 0.05) (Figure 5a,b). For the Simpson index, there were no significant differences in the control group, DSS group *L. acidophilus* NCFM, CCFM137, and FAHWH11L56 groups; however, *L. acidophilus* FGSYC48L79 could significantly decrease the Simpson index (Figure 5c). For the β-diversity, the gut microbiota of the mice in the *L. acidophilus* FGSYC48L79 group were completely different from the other four groups (Figure 5d).

### 2.5. L. acidophilus Modified the Composition of Gut Microbiota in DSS-Induced Colitis Mice

**Figure 6:**
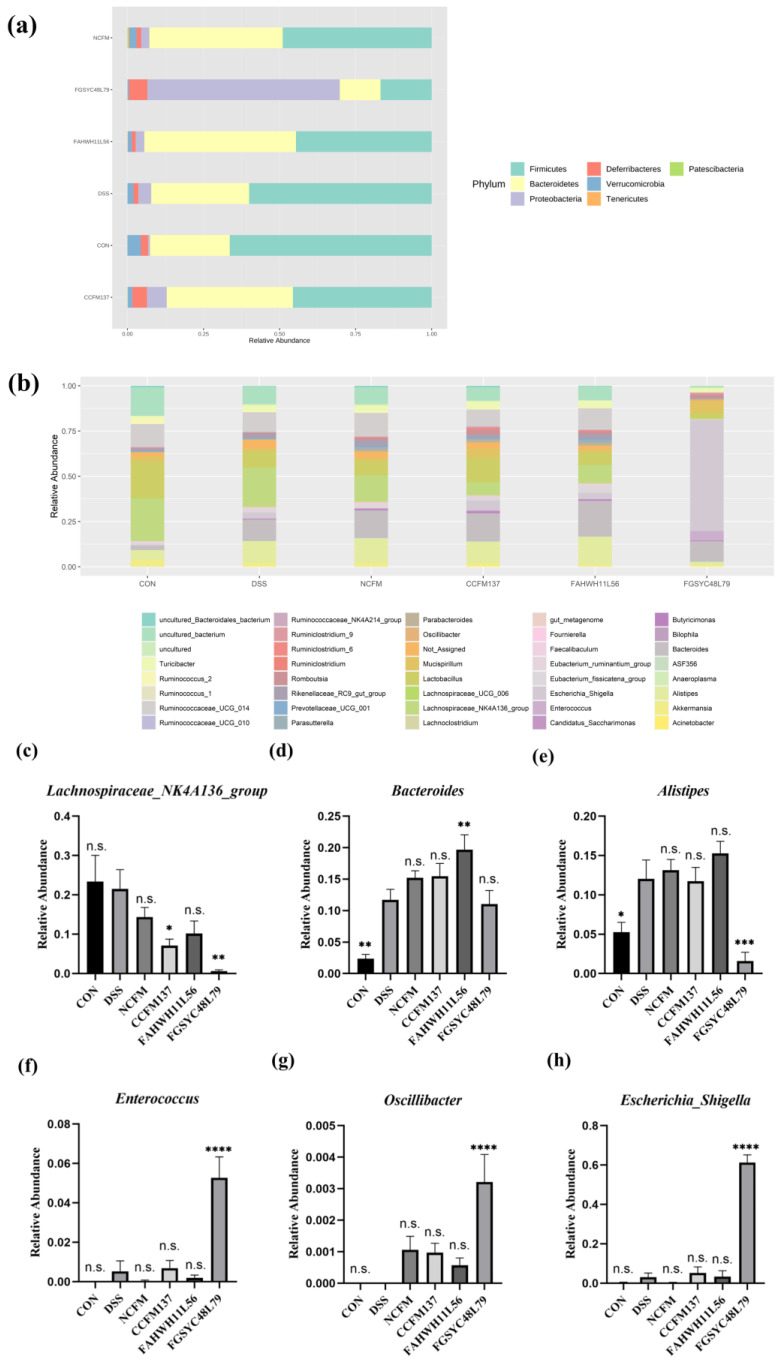
Effects of *L. acidophilus* on the composition of gut microbiota. (**a**) Phylum of gut microbiota, (**b**) genus of gut microbiota, and (**c**–**h**) relative abundance of *Lachnospiraceae* NK4A136 group, *Bacteroides*, *Alistipes*, *Enterococcus*, *Oscillibacter*, and *Escherichia*_*Shigella*. *: *p* < 0.05. **: *p* < 0.01. ***: *p* < 0.001. ****: *p* < 0.0001. N.s.: no significant difference. All data are presented as mean ± standard error of the mean (SEM). The DSS group was used as a reference.

At the phylum level, except for the *L. acidophilus* FGSYC48L79 group, whose dominant phylum was Proteobacteria with a more than sixty percent relative abundance, the dominant bacteria in the other groups were Firmicutes and Bacteroidetes. In the control group, the relative abundance of Bacteroidetes was 26.2%, and the relative abundance of Firmicutes was 66.4%. After the DSS challenge, their relative abundances increased to 32.2% and decreased to 60.0%, respectively, and the relative abundance of Bacteroidetes and Firmicutes after interventions of *L. acidophilus* NCFM, CCFM137, and FAHWH11L56 were 43.8% and 49.0%, 41.5% and 45.6%, and 49.9% and 44.6%, respectively. *L. acidophilus* FGSYC48L79 intervention significantly reduced the relative abundance of these two phyla to 13.4% and 16.8%, respectively (Figure 6a).

At the genus level, the relative abundance of *Lachnospiraceae_NK4A136_group*, a main genus of *Lachnospiraceae*, was the highest in the control group, and its relative abundance was not changed significantly after DSS exposure. Further, *L. acidophilus* CCFM137 and FGSYC48L79 interventions decreased the relative abundance of *Lachnospiraceae_NK4A136_group* significantly (*p* < 0.05) (Figure 6c). In addition, the relative abundance of *Bacteroides* was significantly increased after the DSS challenge (*p* < 0.05). *L. acidophilus* FAHWH11L56 interventions amplified the changes in the relative abundance of *Bacteroides* significantly (*p* < 0.05) (Figure 6d). Moreover, the relative abundance of *Alistipes* also increased significantly after DSS exposure (*p* < 0.05), and *L. acidophilus* NCFM, CCFM137, and FAHWH11L56 did not change its relative abundance significantly compared with the DSS group, while *L. acidophilus* FGSYC48L79 significantly reduced its relative abundance (*p* < 0.05) (Figure 6e). In addition, *L. acidophilus* FGSYC48L79 could significantly increase the relative abundance of *Enterococcus*, *Oscillibacter,* and *Escherchia_Shigella* compared with the DSS group (*p* < 0.05) (Figure 6f–h).

### 2.6. PICRUSt Analysis of Gut Microbiota in DSS-Induced Colitis Mice Modified by L. acidophilus

**Figure 7:**
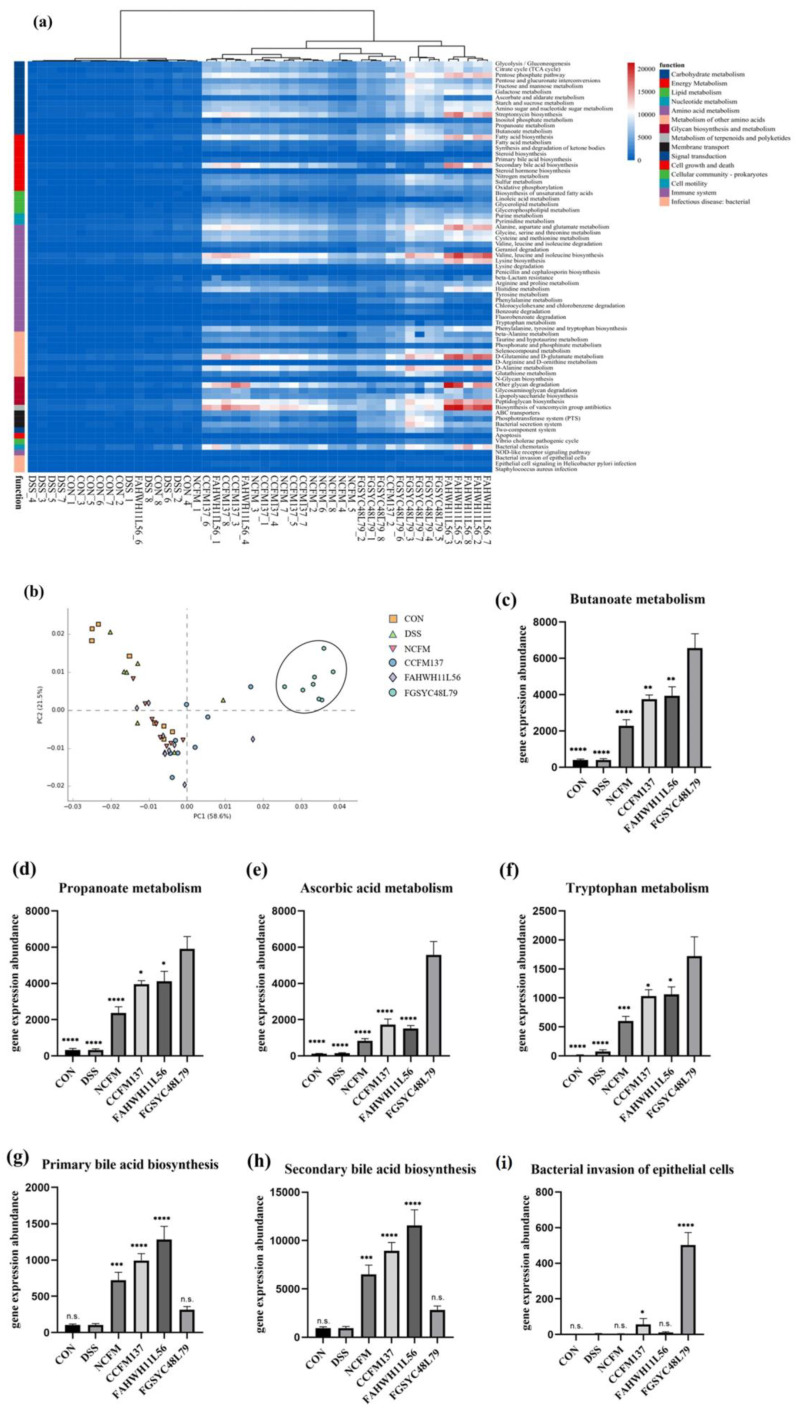
PICRUSt analysis on gut microbiota after *L. acidophilus* intervention. (**a**) Heatmap of prediction on gut microbiota function, (**b**) PCA analysis of prediction on gut microbiota function, (**c**–**i**) predictive gene expression abundance of gut microbiota on butyrate metabolism, propionate metabolism, ascorbate metabolism, tryptophan metabolism, primary bile acid synthesis, and secondary bile acid synthesis of gut microbiota, and ability of bacteria to invade epithelial cells. *: *p* < 0.05. **: *p* < 0.01. ***: *p* < 0.001. ****: *p* < 0.0001. N.s.: no significant difference. All data are presented as mean ± standard error of the mean (SEM). In figure **c**–**f**, the FGSYC48L79 group was used as a reference. In figure **g**–**i**, the DSS group was used as a reference.

The functional changes in gut microbiota in mice with colitis after *L. acidophilus* intervention were predicted by PICRUSt analysis. The results showed that the intervention of *L. acidophilus* had different effects on 15 functional modules, including carbohydrate metabolism, energy metabolism, and lipid metabolism in the gut microbiota of mice with colitis (Figure 7a). The clustering results and PCA results showed that the intervention of *L. acidophilus* FGSYC48L79 significantly changed the function of gut microbiota compared with other groups (Figure 7b).

The prediction results showed that the expression of butyrate metabolism, propionate metabolism, and ascorbic acid metabolism in the gut microbiota of mice after the intervention of *L. acidophilus* FGSYC48L79 was significantly up-regulated compared with other strains (*p* < 0.05) (Figure 7c–f). In addition, *L. acidophilus* FGSYC48L79 also significantly up-regulated the gene expression abundance of bacterial invading epithelial cells in the gut microbiota (*p* < 0.05) (Figure 7i). *L. acidophilus* NCFM, CCFM137, and FAHWH11L56 all significantly increased the expression abundance of genes for primary bile acid synthesis and secondary bile acid synthesis in the gut microbiota (*p* < 0.05) (Figure 7g,h).

### 2.7. L. acidophilus Modified the Fecal SCFA in DSS-Induced Colitis Mice

**Figure 8:**
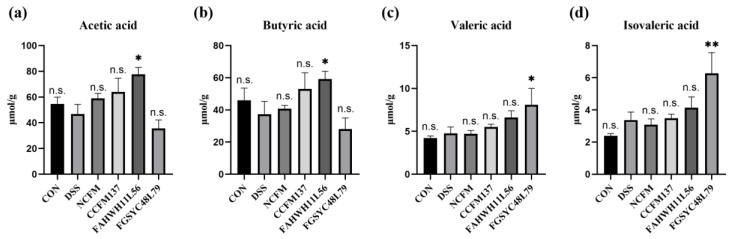
Effects of *L. acidophilus* on the content of short-chain fatty acids. (**a**) Acetic acid, (**b**) butyric acid, (**c**) valeric acid, (**d**) isovaleric acid. *: *p* < 0.05. **: *p* < 0.01. N.s.: no significant difference. All data are presented as mean ± standard error of the mean (SEM). The DSS group was used as a reference.

Fecal SCFAs, including acetic acid, propionic acid, butyric acid, valeric acid, and isovaleric acid, were analyzed. The concentration of propionic acid, valeric acid, and isovaleric acid, showed no significant difference among the control, DSS, *L. acidophilus* NCFM, and *L. acidophilus* FAHWH11L56 groups. For acetic acid and butyric acid, compared with the DSS group, gavage of *L. acidophilus* FAHWH11L56 was shown to be able to significantly increase their level in feces (*p* < 0.05) (Figure 8a,b). In addition, *L. acidophilus* FGSYC48L79 was shown to be able to significantly increase valeric acid and isovaleric acid in feces (*p* < 0.05) (Figure 8c,d).

### 2.8. Genome Analysis of L. acidophilus

**Figure 9:**
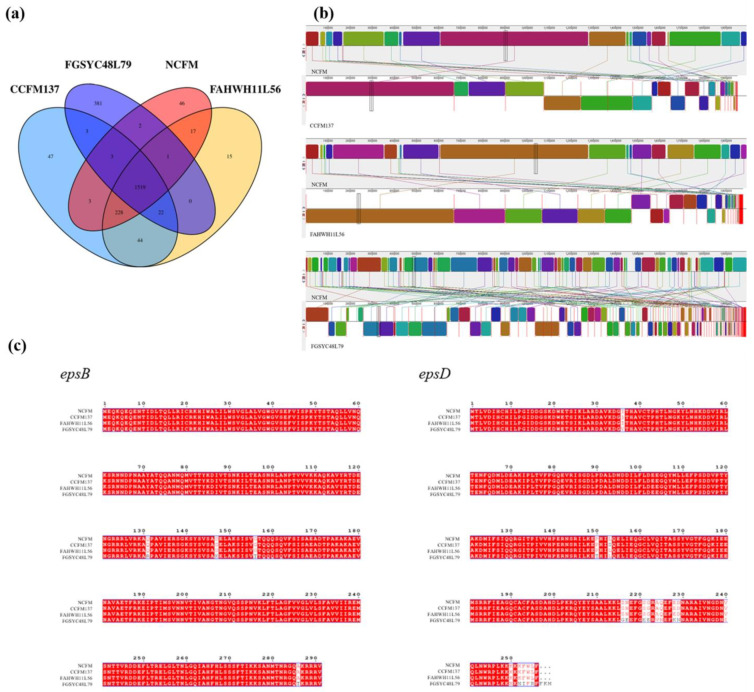
Genomic analysis of *L. acidophilus* NCFM, CCFM137, FAHWH11L56, and FGSYC48L79. (**a**) Venn diagram of *L. acidophilus* based on homologous genes, (**b**) the multiple genome alignment of *L. acidophilus*, and (**c**) amino acid of *epsB* and *epsD* multiple sequence alignment of *L. acidophilus*.

Combined with the results of animal experiments, some genomic analysis was performed to explore the correlation between the genotype and its function in regulating colitis for *L. acidophilus*. From the genome perspective, the number of unique genes of *L. acidophilus* FGSYC48L79 was much higher than those of the other *L. acidophilus* strains (Figure 9a). In addition, a multiple genome alignments analysis was performed on these strains to explore the consistency of their gene numbers and sequences. The results showed that a high level of synteny exists among *L. acidophilus* NCFM, CCFM137, and FAHWH11L56. However, the collinear sequence fragments between the genomes of *L. acidophilus* FGSYC48L79 and the other strains were short and scattered, and there were more genomic re-arrangements and blank areas, which showed that it was more different from other strains (Figure 9b).

The amino acid sequences alignment to the *epsB* and *epsD* genes of *L. acidophilus* NCFM, CCFM137, FAHWH11L56, and FGSYC48L79 were subjected to multiple sequence alignment, and it was found that the amino acid sequences of the *epsB* genes of the four strains were completely consistent, while there were differences in four amino acid sites of *L. acidophilus* FGSYC48L79. *L. acidophilus* NCFM and FAHWH11L56 were completely consistent on the amino acid sequence of the *epsD* gene, while *L. acidophilus* CCFM137 had one different amino acid site compared with *L. acidophilus* NCFM. Of note, the amino acid sequence of *epsD* gene in *L. acidophilus* FGSYC48L79 showed 18 differences from that in *L. acidophilus* NCFM (Figure 9c).

## 3. Discussion

In this study, the effects of several *L. acidophilus* strains on DSS-induced colitis were compared. *L. acidophilus* CCFM137 and FAHWH11L56 showed potential for relieving colitis. Conversely, *L. acidophilus* FGSYC48L79 exacerbated the symptoms of colitis. In previous studies, it had been found that *L. crispatus* CCTCC M206119 aggravated colitis in mice and further damaged the intestinal barrier [10]; *Anaerostipes hadrus* BPB5, a candidate probiotic strain, aggravated the disease activity index and mortality in DSS-induced colitis mice [11]. Such results alert us that some probiotics may not exert positive effects on individuals with disease or gut microbiota imbalance. Therefore, the effects of probiotics in unhealthy individuals should be considered when evaluating the safety of candidate probiotics.

Various cytokines play important roles in colitis, in which IL-1β and TNF-α are important inflammatory cytokines and were expressed abundantly during the period of colitis. The use of antagonists corresponding to these two cytokines can effectively alleviate the symptoms of colitis [12,13]. However, in this study, these two cytokines were not significantly changed, which differed from a previous report that *L. acidophilus* can down-regulate IL-1β and TNF-α to improve colitis [6]. It is speculated that *L. acidophilus*, which alleviated colitis in this research, does not completely change and eliminate the colon inflammation in mice; thus, mice are still in the inflammatory regulation period and require a certain level of inflammatory factors to activate the immunity.

In various cytokines related to colitis, IL-10 is a pleiotropic cytokine that could inhibit NF-κB signaling pathways in the process of inflammation to alleviate chronic inflammatory diseases [14]. *L. acidophilus* FAHWH11L56 could significantly increase IL-10 in this study, and this result was consistent with previous research that the other two *L. acidophilus* strains could also increase IL-10 in colitis mice [5]. Additionally, IL-17 is a central player in immunity and plays an important role in host defense. IL-17 is particularly critical in the epithelial barrier, as it can induce the expression of important pro-inflammatory cytokines, enhance the expression of chemokines, and recruit immune cells by inducing a variety of matrix metalloproteinases [15]. Generally, *Lactobacillus* could inhibit the secretion of IL-17, which plays a role of promoting intestinal inflammation when it relieves colitis [7,16]. However, there have been some studies which suggested that IL-17 can exert protective effects rather than detrimental effects [17], and that it is a protection effector against the adherent-invasive *Escherichia coli* in murine colitis [18]. In the current study, *L. acidophilus* FAHWH11L56 significantly up-regulated IL-17, which might prove that the up-regulation of IL-17 is beneficial during the remission of colitis. CCL2 is a member of the C-C chemokine family, which can regulate the recruitment of myeloid cells. In colorectal cancer, it is closely related to the number of tumors in the colon of mice [19] and the level of CCL2 increases in patients with CRC [20]. CCR2 is the key functional receptor for CCL2, and the CCL2/CCR2 axis is an important pathway of migration of immune cells. It has been a hotspot in inflammation-related diseases research, such as research on pancreatic cancer [21], liver cancer [22], and prostate cancer [23], and CCR2 antagonist can effectively alleviate the related diseases mediated by CCR2. CCL3/CCR1 is another pathway that is related to immune cells migration, inflammatory activation, immune responses, and tumor growth [24,25,26]. In this study, *L. acidophilus* FAHWH11L56 caused a significant increase of CCL2 and CCL3, and a significant decrease of CCR2 and CCR1. Therefore, blocking the signal transmission in CCL2/CCR2 axis and CCL3/CCR1 axis might be an important method for *L. acidophilus* FAHWH11L56 to potentially relieve colitis. Different from *L. acidophilus* FAHWH11L56, *L. acidophilus* CCFM137, although it did not significantly affect the level of IL-17, could reduce the expression of CCL2 and CCL3, and reduce the expression of CCR2 and CCR1. It was able to block the signaling of the CCL2/CCR2 axis and the CCL3/CCR1 axis more completely. For *L. acidophilus* FGSYC48L79, although it blocked the CCL2/CCR2 axis, it can promote the signal transmission on the CCL3/CCR1 axis, which may be one of the reasons why this strain appeared to have the effect of worsening colitis.

Probiotics also affect mice with colitis by regulating the unbalanced gut microbiota. In general, the proportion of Firmicutes decreases in the setting of colitis, which is consistent with a previous report [27]. Further, when an individual has an immune disorder, the number of Proteobacteria, which are originally in a low abundance, begins to increase, which in turn promotes intestinal inflammation [28]. The relative abundance change difference in Proteobacteria may be the reason for the different regulatory effects of different *L. acidophilus* strains.

At the genus level, in this study, the relative abundance of *Lachnospiraceae_NK4A136_group,* the main genus of *Lachnospiraceae*, was increased after DSS challenge, which is consistent with a previous report [29]. A previous study showed that patients with UC had an increased abundance of *Lachnospiraceae* compared to healthy individuals [30]. However, it was also reported that the relative abundance of *Lachnospiraceae* was decreased in IBD patients [31]. *Lachnospiraceae*, one of the core gut microbiota that colonizes the gut from birth and is also one of the major of SCFAs producers, has also been implicated in various intestinal diseases [31]. Therefore, whether *Lachnospiraceae* or *Lachnospiraceae_NK4A136_group* can be used as a biomarker for colitis remains to be further investigated. In this study, its relative abundance was decreased after *L. acidophilus* FAHWH11L56 intervention; this result was similar to the intervention of Jinxiang garlic (*Allium sativum* L.) polysaccharides on DSS-induced colitis [29]. Current studies suggested that *Alistipes* may be protective against colitis [32]. *L. acidophilus* FGSYC48L79 significantly down-regulated its abundance, which may be one of the reasons for its aggravation of colitis. An increased relative abundance of *Enterococcus* spp., predominantly *E. faecalis*, increases the intestinal inflammatory damage [33]. *Oscillibacter*, a newly discovered genus associated with digestive diseases, exacerbates DSS-induced colitis [34]. *Escherchia_Shigella* is a ubiquitous genus of pathogenic bacteria in patients with colitis and colorectal cancer [35]. However, *L. acidophilus* FGSYC48L79 significantly up-regulated the relative abundance of these three genera, which would lead to the aggravation of colitis.

Treatment of *L. acidophilus* changed the composition of gut microbiota and, through further analysis of gut microbiota, it was found that the functional prediction of the gut microbiota was changed. Butyric acid, propionic acid, ascorbic acid [36], and tryptophan [37] are reported to have the capacity to improve colitis. For the four metabolites, *L. acidophilus* FGSYC48L79 may up-regulate the metabolic capacity of gut microbiota, which may decrease their concentrations in the colon hence, this may be one of the reasons for its failure to relieve colitis. *L. acidophilus* FGSYC48L79 may also cause harmful bacteria to break through the intestinal barrier and affect the body negatively. Bile acid dysbiosis occurs in patients with inflammatory bowel disease [38]. Secondary bile acids can exert anti-inflammatory activity in the gut [39], and *L. acidophilus* NCFM has been reported to have the ability to affect intestinal bile acids [40]. In this study, *L. acidophilus* CCFM137 and FAHWH11L56 showed potential for relieving colitis, and can significantly increase the gene expression of primary bile acid synthesis and secondary bile acid synthesis in the gut microbiota.

Changes in gut microbiota will inevitably lead to changes in the SCFAs. As main products produced by gut microbes, SCFAs are the modulators of colonic function and inflammatory response [41,42]. The supplementation of acetate or butyrate is a proven method to exert anti-inflammatory effects in individuals [43,44]. This suggests that the significant increase in acetic acid and butyric acid by *L. acidophilus* FAHWH11L56 treatment may also be the reason for its potential for relieving colitis. Although *L. acidophilus* FGSYC48L79 can significantly increase the levels of valeric acid and isovaleric acid, it did not have relieve colitis. It was speculated that the improving level of valeric acid and isovaleric acid brought by *L. acidophilus* FGSYC48L79 cannot offset its negative effects on colitis.

Genetic analysis of these four strains was performed in this study to preliminarily explore their genetic and functional connections. Based on previous research on comparative genomics of *L. acidophilus* in our laboratory [9] and the new results in this study, in short, *L. acidophilus* FGSYC48L79 showed a longer evolutionary distance and a higher genomic uniqueness with other three strains used in this study. The overall genomic differences indicated that there may be large differences in the functions of the strains; however, the specific functional performance needs to be further analyzed in combination with specific functional genes.

It was suggested that probiotic exopolysaccharides could exert immunomodulatory activity in colitis [45], including the exopolysaccharides from *L. acidophilus* [46]. The known exopolysaccharide synthesis genes of other lactic acid bacteria, the *epsB* and *epsD* genes, may be the main genetic determinants for exopolysaccharides synthesis in *L. acidophilus*. Based on previous research on comparative genomics of *L. acidophilus* in our laboratory, *L. acidophilus* FGSYC48L79 lost many genes in the exopolysaccharide gene cluster [9]. In this study, *L. acidophilus* FGSYC48L79 showed differences from other *L. acidophilus* in both *epsB* and *epsD* genes. However, how these differences on exopolysaccharide gene cluster affect the production and function of exopolysaccharides is still not clear, and needs to be further experimentally verified. For *L. acidophilus* NCFM, CCFM137, and FAHWH11L56, it can only be hypothesized that their exopolysaccharide gene clusters are relatively complete and, thus, they can synthesize exopolysaccharides and have a potential relieving effect on colitis.

Despite exopolysaccharides, surface protein A also exerts immunomodulatory activity in colitis [4] but is regulated by a single gene. Based on our previous research on comparative genomics of *L. acidophilus* [9], the deletion of the *slpA* gene in *L. acidophilus* FGSYC48L79 may be one of the reasons for its failure to alleviate colitis. For the other three *L. acidophilus* strains, *L. acidophilus* NCFM and FAHWH11L56, which contained the *slpA* gene, had commonality in affecting cytokines and chemokines, while *L. acidophilus* CCFM137, a strain which has lost the *slpA* gene, was different from the other two strains in affecting cytokines and chemokines; hence, it is speculated that the *slpA* gene may not be a key point for *L. acidophilus* CCFM137 to show potential for relieving colitis.

Carbohydrates are the main source of nutrition for the gut microbiota, and the structure of the gut microbiota changes when it is disturbed differently [47]. Based on our previous research [9], the carbohydrate transport and metabolism genes in *L. acidophilus* FGSYC48L79 were significantly different from those in *L. acidophilus* NCFM, CCFM137, and FAHWH11L56, although the same rearing conditions led to huge differences in gut microbiota changes. At the same time, *L. acidophilus* FGSYC48L79 showed different genes in energy production and amino acid transport, which may also have an impact on the gut microbiota. Therefore, the increased relative abundance of harmful bacteria in the gut microbiota of mice after *L. acidophilus* FGSYC48L79 intervention may be related to the aforemntioned differences.

## 4. Materials and Methods

### 4.1. Bacterial Strains and Preparation

*L. acidophilus* CCFM137, *L. acidophilus* FAHWH11L56 (CCFM1200), and *L. acidophilus* FGSYC48L79 were deposited at the Culture Collection of Food Microorganisms in Jiangnan University, Wuxi, China (CCFM), and their genomes were sequenced and uploaded to GenBank with accession number PRJNA736624 (https://www.ncbi.nlm.nih.gov/bioproject/736624) (accessed on 10 June 2022). *L. acidophilus* NCFM was used as a positive control [8]. All the strains were cultured in de Man, Rogosa, and Sharpe (MRS) medium at 37 °C for 24 h under an anaerobic environment (AW500SG, Electrotek Scientific Ltd., West Yorkshire, UK) flushed with 10% hydrogen, 10% carbon dioxide, and 80% nitrogen. All the bacteria were harvested by centrifuging at 8000 g for 15 min at 4 °C and stored at 4 °C in glycerol before use. The number of viable bacteria cells was adjusted to be 1 × 10^9^ CFU/mL with sterile saline for each strain for the animal trial.

### 4.2. Animal Trial Design

Six-week male specific pathogen-free (SPF) C57BL/6N mice were purchased from Vital River Laboratory Animal Technology Company Co., Ltd. (Shanghai, China). All the procedures were approved by the Experimental Animal Management and Animal Welfare Ethics Committee of Jiangnan University (JN.No20210415c1360601[071]). Mice were housed in an SPF environment at 20 ± 2 °C and relative 55 ± 5% humidity with monitored light (light, 12 h; dark, 12 h) and with free access to sterile water and food.

After one-week adaptation, a total of 48 male mice was randomly divided into six groups including the control group, DSS group, *L. acidophilus* NCFM group (Positive group), *L. acidophilus* CCFM137 group, *L. acidophilus* FAHWH11L56 group, and *L. acidophilus* FGSYC48L79 group. During the two weeks of the trial, mice in the control and DSS groups were orally gavaged with sterile saline (200 μL per mouse) daily, while mice in all the four *L*. *acidophilus* groups were administrated with the corresponding bacterial suspension (200 μL per mouse) daily, respectively. In the second week, 2.5% (*w*/*v*) DSS (molecular weight 36,000–50,000, MP Biomedicals, LLC, Irvine, CA, USA) was added to the water for the mice, for all the groups except the control mice.

### 4.3. Assessment of Colitis Symptoms

During the DSS exposure, the body weight and disease activity index (DAI) of each mouse were monitored daily. When the mice were euthanized, their colon length was measured. Colon tissues were dehydrated, embedded, sliced, and stained with hematoxylin and eosin (H&E) [48].

### 4.4. Biochemical Assays

The colon tissues were collected and homogenized in PBS, and then centrifuged to remove impurities. The changes in interleukin (IL)-1β, IL-10, IL-17, and tumor necrosis factor (TNF)-α were measured by enzyme-linked immunosorbent (ELISA) kit according to the manuals (R&D Systems, Inc. Minneapolis (MPLS), MN, USA). 

### 4.5. RNA Isolation and Real-Time Quantitative-PCR

Trizol Reagent and HiScript^®^ III RT SuperMix for qPCR (Vazyme Biotech Co., Ltd., Nanjing, China) were used to extract total RNA and reverse total RNA into cDNA. Real-time PCR was performed using the ChamQ Universal SYBR qPCR Master Mix (Vazyme Biotech Co., Ltd., Nanjing, China) with a CFX384 Touch real-time PCR detection system (Bio-Rad Co., Ltd., Hercules, CA, USA). Sequences of primer are shown in Table 1. The relative expression of β-actin was determined with the 2^−ΔΔCt^ method.

### 4.6. DNA Extraction and Pyrosequencing

Before mice were sacrificed, fresh fecal samples were collected. Bacterial DNA of fresh fecal samples was extracted with a FastDNA SPIN kit (MP Biomedicals, LLC, Irvine, CA). A universally bacterial primers pair, 341F (5′-CCTAYGGGRBGCASCAG-3′) and 806R (5′-GGACTACNNGGGTATCTAAT-3′), was used to amplify the V3-V4 region of 16S ribosomal RNA (rRNA) gene as previously described [49]. PCR products were purified using a QIAquick gel extraction kit (Qiagen GmbH, Hilden, Germany) and quantified according to the manual. The purified PCR products were sequenced (2 × 300 bp) on an Illumina MiSeq platform (Illumina Inc., San Diego, CA, USA) as previously described [50]. The sequenced data were processed using the QIIME 2 pipeline [51]. MicrobiomeAnalyst platform (https://www.microbiomeanalyst.ca/faces/home.xhtml?tdsourcetag=s_pcqq_aiomsg) (accessed on 19 June 2022) was used to perform data analysis of gut microbiota. PICRUSt2 was used to predict the function of gut microbiota [52].

### 4.7. Short Chain Fatty Acid Determination

The extraction of fecal SCFAs was performed as previously described [53]. The extracted SCFAs were measured by gas chromatography (GC)—mass spectrometry (MS) with previously-described parameters [53].

### 4.8. Genome Analysis

The genome of *L. acidophilus* NCFM is available on the National Centre for Biotechnology Information (NCBI) website (https://www.ncbi.nlm.nih.gov/) (accessed on 10 June 2022). The genome data of other strains analysed in this study were submitted to the NCBI Sequence Read Archive (https://www.ncbi.nlm.nih.gov/sra/) (accessed on 10 June 2022) in our previous study under the BioProject PRJNA736624. The venn diagram of *L. acidophilus* strains was made by using Orthomcl software [54]. MAUVE software was used to perform multiple genome alignment. ESPript Web server was used to analyze multiple sequence alignment of amino acid [55].

### 4.9. Statistical Analysis

Graphpad Prism 8.0.2 was used for statistical analysis, and the significant difference was evaluated using One-Way ANOVA for multiple comparisons. All the results were expressed as means ± standard error of the mean, and the statistically significant difference was expressed as *p* < 0.05. The correlation analysis of gut microbiota and SCFAs was performed using Hiplot software and Graphpad Prism 8.0.2. The original data set for GEO data analysis was downloaded from GEO on NCBI, the serial accession number of the original data was GSE22307, and its KEGG pathway enrichment data processing was done by Hiplot.

## 5. Conclusions

*L. acidophiluss* CCFM137 and FAHWH11L56 showed potential for relieving colitis, and this may be related to their potential for regulating cytokines, regulating short-chain fatty acids, regulating gut microbiota and their functions, and blocking the signaling of chemokines and their receptors. In contrast, *L. acidophilus* FGSYC48L79 exacerbated colitis, possibly by increasing the abundance of harmful bacteria in the gut microbiota while altering gut microbiota function and promoting signaling of cellular chemokines and their receptors. The different effects of *L. acidophilus* on colitis may be related to the genotypic differences in various functional genes.

## Figures and Tables

**Table 1:** Sequences of primers.

Primer	Sequence
CCL2-F	5′-TTAAAAACCTGGATCGGAACCAA-3′
CCL2-R	5′-GCATTAGCTTCAGATTTACGGGT-3′
CCL3-F	5′-TTCTCTGTACCATGACACTCTGC-3′
CCL3-R	5′-CGTGGAATCTTCCGGCTGTAG-3′
CCR2-F	5′-ATCCACGGCATACTATCAACATC-3′
CCR2-R	5′-CAAGGCTCACCATCATCGTAG-3′
CCR1-F	5′-CTCATGCAGCATAGGAGGCTT-3′
CCR1-R	5′-ACATGGCATCACCAAAAATCCA-3′

## Data Availability

All raw sequencing data analysed in this study have been submitted to the NCBI Sequence Read Archive (https://www.ncbi.nlm.nih.gov/sra/) (accessed on 10 June 2022) under the BioProject PRJNA736624.
